# Balancing Dopamine: Managing Parkinson’s Disease and Psychiatric Symptoms through Agonist-Antagonist Pharmacotherapy

**DOI:** 10.1192/j.eurpsy.2025.814

**Published:** 2025-08-26

**Authors:** J. C. Hall, G. Legerme, D. Nolasco, A. Wu, B. Carr

**Affiliations:** 1 College of Medicine; 2 Department of Psychiatry, University of Florida, Gainesville, United States

## Abstract

**Introduction:**

Parkinson’s disease (PD) is characterized by basal ganglia dopamine depletion, leading to motor symptoms. Psychiatric symptoms, like psychosis, are associated with dopamine overactivity in the mesolimbic and mesocortical system. PD patients sometimes require both motor and psychiatric treatment. However, the pharmacologic treatments for each have opposing dopaminergic effects. Understanding the balance between dopamine antagonists to treat psychosis while increasing dopamine agonists (DAs) to treat PD is a difficult clinical task.

**Objectives:**

We aim to explore how dopamine agonists and antagonists affect the responsiveness of dopamine receptors in various brain regions.

**Methods:**

We conducted a comprehensive literature review on pharmacological management of patients with PD and concurrent psychiatric symptoms. Special attention was given to balancing dopamine agonists (e.g., Carbidopa-Levodopa) for PD and dopamine antagonists (e.g., Quetiapine) for psychiatric symptoms.

**Results:**

While dopamine agonists and antagonists appear counterintuitive when used concurrently, their efficacy is contingent on target brain regions. DAs (Carbidopa-Levodopa) are most beneficial for increasing dopamine deficits in the striatum and nigrostriatal pathway, where voluntary movement is controlled. In PD, this pathway is primarily affected, and DAs are used to target the striatum’s high concentration of D1 and D2 sub-receptors. Gold-standard DAs mainly target D1 and D2 receptors.

Dopamine antagonists mitigate excess dopamine activity in the mesolimbic and mesocortical systems, which affect reward, memory, and executive functioning. These systems possess a high concentration of D3 and D4 sub-receptors. Typical antipsychotics have strong D2 receptor affinity, making them counterintuitive to DA motor treatment. Atypical antipsychotics have partial D2 affinity, but also readily bind D3 and D4. Some atypicals minimize D1/D2 affinity to allow DAs to be more effective. Quetiapine is currently used for psychosis in PD, but the drug’s MOA is not fully understood. Cariprazine binds D3 well but does cause extrapyramidal effects with its D2 affinity. Clozapine strongly binds D4 but can have severe adverse effects. Additionally, pramipexole and ropinirole have strong D3 and D4 affinity. Ultimately, the key lies in symptom control: achieving optimal motor function while controlling psychiatric manifestations.

**Conclusions:**

PD management with concurrent psychiatric disease requires diligent pharmacologic balance of countering dopamine treatments. Dopamine agonists and dopamine antagonists do have opposed pharmacodynamics, but clinicians must understand that certain pharmacologic agents do not all target the same brain area nor dopamine sub-receptor. Clinicians must use this pharmacologic knowledge to carefully balance these therapies by adapting to the individual patient’s symptomatology and treatment response.

**Disclosure of Interest:**

None Declared

